# Clinical Applications of Optical Coherence Tomography Angiography in Inherited Retinal Diseases: An Up-to-Date Review of the Literature

**DOI:** 10.3390/jcm12093170

**Published:** 2023-04-28

**Authors:** Claudio Iovino, Clemente Maria Iodice, Danila Pisani, Luciana Damiano, Valentina Di Iorio, Francesco Testa, Francesca Simonelli

**Affiliations:** Eye Clinic, Multidisciplinary Department of Medical, Surgical and Dental Sciences, University of Campania Luigi Vanvitelli, 80131 Naples, Italy

**Keywords:** Best disease, choroideremia, inherited retinal diseases, optical coherence tomography angiography, retinitis pigmentosa, Stargardt disease

## Abstract

Optical coherence tomography angiography (OCT-A) is a valuable imaging technique, allowing non-invasive, depth-resolved, motion-contrast, high-resolution images of both retinal and choroidal vascular networks. The imaging capabilities of OCT-A have enhanced our understanding of the retinal and choroidal alterations that occur in inherited retinal diseases (IRDs), a group of clinically and genetically heterogeneous disorders that may be complicated by several vascular conditions requiring a prompt diagnosis. In this review, we aimed to comprehensively summarize all clinical applications of OCT-A in the diagnosis and management of IRDs, highlighting significant vascular findings on retinitis pigmentosa, Stargardt disease, choroideremia, Best disease and other less common forms of retinal dystrophies. All advantages and limitations of this novel imaging modality will be also discussed.

## 1. Introduction

Optical coherence tomography angiography (OCT-A) is a novel imaging technique that relies on the intrinsic movement of red blood cells (RBCs), allowing non-invasive, motion-contrast, high-resolution images of both retinal and choroidal vascular networks [1].

The retina is supplied by up to 4 layers of vessels: (1) the radial peripapillary capillary network, within the nerve fiber layer and located around the optic nerve head; (2) the superficial vascular plexus, within the ganglion cells layer; (3) the deep capillary complex, which comprises 2 capillary beds on both sides of the inner nuclear layer [2].

The choroid, conversely, consists of 3 layers of vessels: (1) the Haller layer, the outer, large-caliber layer of vessels; (2) the Sattler layer, the middle, smaller-diameter layer of vessels; (3) the choriocapillaris, which is the innermost and smallest layer of vessels [2].

OCT-A is able to clearly display several vascular alterations, including, among others, areas of macular telangiectasia, impaired perfusion, microaneurysms, capillary remodeling and neovascularization [3]. In contrast with conventional imaging modalities, the dye-free image acquisition of this method avoids the onset of typical side effects of fluorescein and indocyanine green angiography (FA and ICGA) [4,5].

More importantly, OCT-A allows depth-resolved analysis of retinal tissue that has never been available before [3]. OCT-A has been adopted to investigate a broad spectrum of retinal vascular diseases, ranging from diabetic retinopathy and retinal venous occlusion, up to age-related macular degeneration, and inflammatory and ocular oncology disorders [3]. Over the past 15 years, the retinal and choroidal imaging capabilities of OCT-A have been applied to further characterize primary and secondary alterations in inherited retinal diseases (IRDs). In this review of the literature, we aim to analyze and summarize all clinical applications of OCT-A in the diagnosis and management of IRDs and to discuss advantages and limitations of this imaging technique.

## 2. Optical Coherence Tomography Angiography Technical Aspects

OCT-A is an optical coherence tomography (OCT)-based imaging technique that enables the visualization of blood vessels within the eye, and it is built on the principle of OCT signal variation generated by the moving RBCs within the vessels [6,7,8]. Multiple scans are performed at the same location and the subsequent temporal changes of the OCT signal caused by the constant motion of the RBCs generate angiographic contrast, allowing visualization of the microvasculature [3].

Barton et al., in 2005, laid the foundation for this relatively new technology, which has only been commercially available since 2016 [9]. The authors adjusted analysis of speckles to produce an amplitude-based angiogram [9]. The speckle pattern stays relatively constant over time for static objects, while it changes for moving scatterers (i.e., erythrocytes) [9]. In 2009, Wang et al. introduced optical microangiography (OMAG), an imaging technique in which spatial frequency analysis of time-varying spectral interferograms was used to distinguish the signals backscattered by particles in motion from those backscattered by static objects, creating a high-resolution angiogram image [10]. Subsequently, in 2012, Jia et al. developed a more refined signal processing algorithm, named split-spectrum amplitude-decorrelation angiography (SSADA), which enhanced the signal-to-noise ratio of flow detection while reducing the pulsatile bulk-motion noise [11].

OCT-A may be captured with spectral domain OCT (SD-OCT), which, in commercial devices, employs a wavelength of ~840 nm, or with swept-source OCT (SS-OCT), which uses a longer wavelength of ~1050 nm [12].

While OCT is considered a cross-sectional imaging modality, OCT-A images are mainly studied with en face visualization. Currently, all commercially available OCT-A platforms allow the segmentation of the volumetric scans at specific depths through the definition of “slabs” [12].

FA and ICGA have been considered, so far, the gold standard for the evaluation of retinal and choroidal vasculature in vivo. Nevertheless, although dye injection is generally safe, serious allergic reactions may occur and these techniques are therefore considered invasive [12]. Moreover, the use of dyes in pregnant or breastfeeding women appears to be controversial [13,14].

OCT-A provides a non-invasive and fast analysis of choroidal and retinal microvascular circulation without the need for any dye injection. Moreover, it has the additional advantage of depth-resolution with better visualization of the deeper vascular layers [12].

## 3. Clinical Applications

### 3.1. OCT-A in Retinitis Pigmentosa

Most of the literature about the findings of OCT-A in retinitis pigmentosa (RP) converged to a common demonstration of retinal and choroidal vascular impairment. A summary of the data collected is reported in Table 1.

The mean follow-up ranged between 2 months and 36 months [12,13,14,15,16,17,18,19,20,21,22,23,24,25,26,27,28,29,30,31]. Overall, significant reductions in both the superficial capillary plexus (SCP) and deep capillary plexus (DCP) were observed in all the affected patients of the evaluated cohorts over time [12,13,14,15,16,17,18,19,20,21,22,23,24,25,26,27,28,29,30,31]. In addition, all the studies that explored the involvement of choriocapillaris (CC) demonstrated its significant impairment in RP patients [12,15,16,17,21,22,23,25,30,31]. Several authors focused on the variation of the foveal avascular zone (FAZ) area in RP patients, two-thirds of which described an increased avascular area [12,15,19,20,26,31], while the remaining third demonstrated its significant reduction [16,17,29]. Nakajima et al. and Alnawaiseh et al. explored an interesting association between the reduction in optic nerve head (ONH) vessel density (VD) in RP patients and the deterioration of the visual field mean deviation (MD) [15,27]. The authors demonstrated that the VD in both the radial peripapillary capillary network and ONH layers was significantly lower in patients rather than controls, significantly correlating with the MD and the cup/disc area ratio [15,27]. Mastropasqua et al. investigated the mean microperimetry (MP) retinal sensitivity between RP patients and healthy subjects and explored possible correlations with retinal perfusion density [25]. The authors found a significant reduction in retinal sensitivity in RP patients, compared to healthy controls, at 4°, 8° and 20° [25]. A significant positive correlation was also observed in RP patients between the perfusion density of the central 1.5 mm retina in either DCP and CC and microperimetry at 4° and 8°, meaning that a reduction in the perfusion density would be associated with a retinal sensitivity decrease [25]. Toto et al. demonstrated instead that parafoveal SCP and DCP VD were significantly correlated with mfERG values, while parafoveal CC VD correlated directly with the P1R2 amplitude, highlighting that vessel impairment may affect macular function [33].

**Figure 1:**
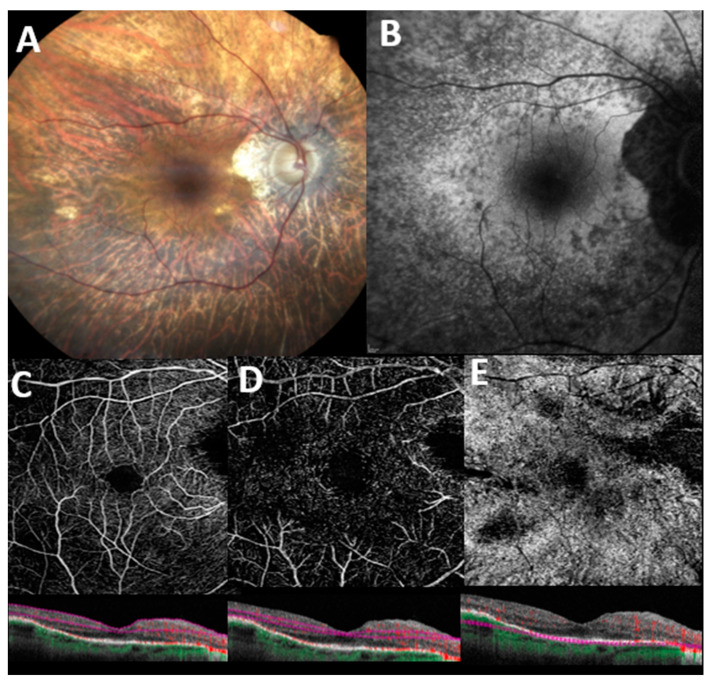
**Multimodal imaging features in a patient with genetically confirmed retinitis pigmentosa**. (**A**) Color fundus image displays pallor of the optic disc, attenuation of retinal vessels, extensive retinal atrophy, and pigmentary clumping in mid-periphery. (**B**) Blue-light autofluorescence (BAF) shows a granular hypoautofluorescence extending from the perifoveal region to the midperiphery. En face 6 × 6 optical coherence tomography angiography with corresponding B scan angio flow of superficial capillary plexus (**C**), deep capillary plexus (**D**), and choriocapillaris (**E**) with automatic segmentation. Flow voids areas are denoted in all retinal plexuses, and especially in the choriocapillaris, possibly related to either segmentation artifacts, outer retinal atrophy, or extremely reduced blood flow which fails to produce a signal (see corresponding B scans angio flow).

A representative case of RP patient examined with OCT-A is shown in Figure 1.

### 3.2. OCT-A in Choroideremia

Following animal model-based studies confirming the primary degeneration of RPE, photoreceptors and CC in choroideremia (CHM), Jain et al. showed, in a 6-month prospective study, that regional changes in CC density correlate with photoreceptor structural alterations in CHM [35,36,37]. They stratified their cohort in 3 groups based on the diagnosis of CHM, CHM carrier state, and healthy controls, demonstrating a significant difference of mean (±SD) CC density among them (82.9% ± 13.4%; 93.0% ± 3.8%; 98.2% ± 1.3%, respectively) [37]. Interestingly, the mean (±SD) CC density in affected eyes was also higher in regions with a preserved, rather than absent, ellipsoid zone (92.6% ± 5.8% vs. 75.9% ± 12.6%, mean difference, 16.7%; 95% CI, 12.1% to 21.3%; *p* < 0.001) [37]. En face outer retinal imaging in these eyes revealed an interesting degeneration pattern with a relatively unaffected central island of photoreceptors showing pseudopodial-like protrusions of surviving tissue, representing scrolled outer retina and outer retinal tubulations (ORTs) at the degeneration margins [38]. The formation of these features suggested that the underlying CC/Retinal pigment epithelium (RPE) would not adequately support the overlying retina, and that photoreceptor death could be a secondary process [37,38].

Abbouda et al. prospectively enrolled 26 eyes, 17 of which had a CHM diagnosis and 9 with a carrier status, focusing on superficial retinal vessel network (SRVN) and CC changes [39]. Both vascular networks appeared significantly reduced in CHM patients if compared to carriers and controls (SRVN: 12.93 ± 2.06 mm^2^, 15.36 ± 0.60 mm^2^, and 15.30 ± 1.35 mm^2^, respectively; CC: 6.97 ± 5.26 mm^2^, 21.65 ± 0.17 mm^2^, and 21.36 ± 0.76 mm^2^, respectively) [39]. Since the presence of a functional CC flow area was positively correlated to the SVRN, the authors postulated that a reduction in CC flow caused a compensatory reduction in SVRN circulation to keep retinal and choroidal circulations balanced [39]. In this regard, again, the missing step between the reduction in CC and SVRN flow could be the expression of reduced metabolic demand of that area of retina, due to the death of either RPE or photoreceptors [37,38].

Battaglia Parodi et al. prospectively examined a consecutive series of 12 eyes of 6 patients with a CHM diagnosis, and compared them with a group of healthy, age-matched controls with no ocular nor systemic disease [40]. The authors found no differences in SCP between cases and controls, both in terms of morphology and vessel density quantification, even by analyzing the preserved central island and external affected area separately [40]. Conversely, a statistically significant impairment was found with regard to DCP and CC. CHM patients displayed a reduced DCP vascular density in both the external macular area (0.017 ± 0.02; *p* < 0.01) and central preserved island (0.037 ± 0.02; *p* < 0.01) compared to controls (0.43 ± 0.03 and 0.43 ± 0.03, respectively) [40]. With regard to CC vessel densities, the peripheral macular area exhibited a significant reduction in patients (0.0 ± 0.0; *p* < 0.01) versus controls (0.49 ± 0.02) while no significant differences were demonstrated in the central preserved island [40]. This finding highlights the coexistence of two CC vessel density patterns, disclosing no changes in correspondence with preserved RPE islands, and an almost undetectable CC vessel density in external regions of substantial RPE deficiency. This supports the current belief that CC loss would occur secondary to RPE loss, not independently [40].

Murro et al. consecutively enrolled 14 eyes of 7 patients with CHM and 14 eyes of 7 healthy controls, demonstrating patients’ significantly smaller FAZ in SCP and DCP (19,899 ± 8368 and 24,398 ± 86,11, respectively) when compared to controls (288,708 ± 4505 and 32,016 ± 4821, respectively) [41]. Quantitative analysis also disclosed statistically significant decreased SCP, DCP, and CC vascular densities, comparing patients with the age-matched control groups [41]. The same authors also explored OCT-A features of 6 CHM carriers (12 eyes), comparing their findings with 8 age-matched controls (16 eyes) [42]. The quantitative analysis of the inner retinal vasculature disclosed no significant differences in both SCP and DCP vessel densities compared to the control group [42]. Only CC showed a mild reduction in the vascular flow in the carrier versus control group (78.896 ± 13.972 vs. 80.008 ± 10.862; *p* = 0.045) [42]. Of note, OCT allowed the identification of the impaired RPE layer in the presence of a preserved central inner retinal and CC vascularization, suggesting that vascular impairment would follow RPE loss in the natural history of the disease [42].

Arrigo et al. designed an observational, cross-sectional clinical series with 7 CHM patients (14 eyes) and 7 age-matched controls (14 eyes), correlating retinal layer thickness with OCT-A findings [43]. Patients displayed significant differences with respect to DCP and CC vascular densities (F = 3941.3 and 655.9, respectively) [43]. Authors also stratified the cohort, assessing the vascular network densities independently based on chorioretinal atrophy areas and anatomically preserved islets [43]. They found that CHM patients displayed significantly lower DCP vascular density in both the atrophic and healthy areas when compared to healthy controls [43]. On the other hand, CC vascular density appeared to be impaired only in the atrophic region (*p* < 0.001) and not in the apparently preserved islet (*p* = 0.19), while SCP was found to be unaffected in both regions (*p* > 0.05) [43]. Interestingly, significant correlations were found between the reduction in DCP vascular density and the thinning of outer plexiform layer, inner nuclear layer, and inner plexiform layer [43].

The utility of OCT-A in the management of choroidal neovascularization (CNV) as a later-stage complication of CHM was also investigated [44,45]. The authors described evidence of a high-flow CC neovascular network in the context of a neighboring vascular attenuation, which regressed to a small juxtafoveal subretinal hyper-reflective lesion after prompt anti-VEGF treatment [44,45].

**Figure 2:**
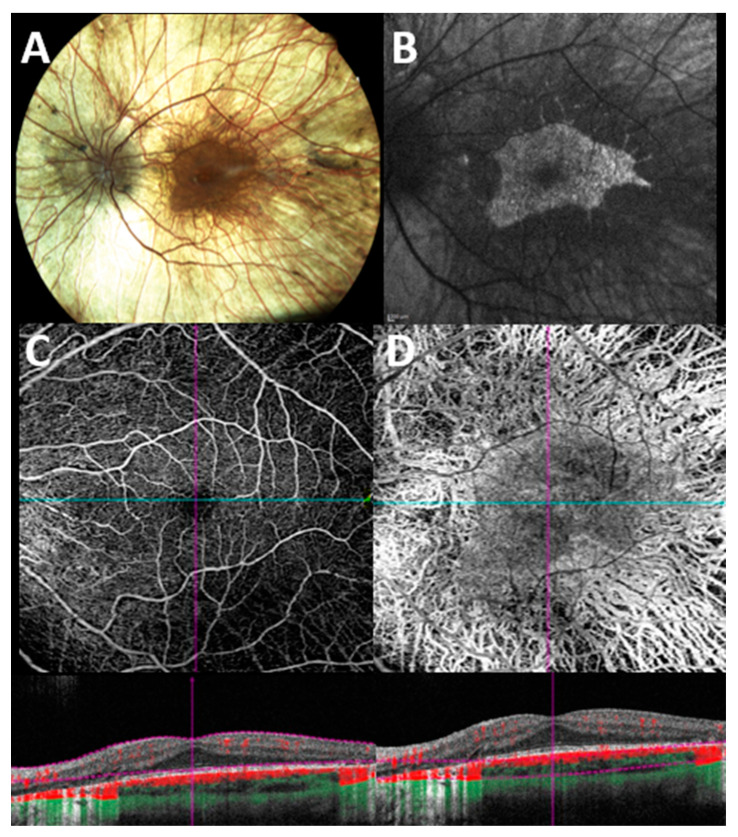
**Multimodal imaging evaluation in a patient with genetically confirmed choroideremia**. (**A**) Color fundus photograph shows extensive retinal degeneration with chorioretinal atrophy. (**B**) Blue light fundus autofluorescence shows typical patterns of a sharply demarcated macular area of remaining tissue (hyper/iso-autofluorescent) against surrounding atrophic RPE (hypoautofluorescent background). (**C**) En face 6 × 6 optical coherence tomography angiography (OCT-A) with corresponding B scan angio flow of the superficial capillary plexus (SCP) shows a preserved macular flow with some areas of flow reduction along the vascular arcade due to the underlying outer retinal atrophy. (**D**) En face 6 × 6 OCT-A with corresponding B scan angio flow of the choroidal slab shows a diffuse loss of vasculature with a relatively preserved island of flow in the foveal region.

A representative case of a patient with CHM examined with OCT-A is shown in Figure 2.

### 3.3. OCT-A in Best Disease

Best vitelliform macular dystrophy (BVMD), also known as Best disease, is an autosomal-dominant inherited disorder caused by mutations in *BEST1* gene [46].

Vascular impairment in Best disease is described in the literature as a later-stage finding in the vitelliform and pseudohypopion stages; the subretinal deposits often cover the CC, showing an OCT-A dark area and, as the deposits disappear and the atrophy progresses, the CC would appear accordingly brighter and more granular [47]. This phase would coincide with the onset of vascular alterations and morphological changes [47]. Likewise, the choroid will change in thickness depending on the stage of the disease, generally appearing thicker in early stages and tending to get thinner in later phases [48].

In the literature, a reduction in vascular flow density is described in SCP and DCP layers, along with a significant FAZ enlargement [49]. Nevertheless, Mirshahi et al. described the presence of a capillary plexus across the FAZ, which could be consequent to a rise in the concentration of angiogenetic factors [50].

Vascular impairment was not only found in retinal vascular layers, but also in the CC. In particular, the CC flow density has been shown to decrease as the disease progresses [48].

Rarely, in about 10% of cases, CNV may occur, leading to a significant loss of vision [51]. Parodi et al. hypothesized a distinct mechanism of neovascularization according to the disease stage [52]. In particular, the early stages (stages 2 and 3) of BVMD are more likely to present with exudative CNV, characterized by higher values of both vessel tortuosity (VT) and vessel dispersion (VDisp) upon OCT-A examination [52]. On the contrary, late stages mainly display non-exudative CNV, with lower perfusion, VT, and VDisp. This would suggest that exudative CNV is associated with a faster growing neovascular network, whereas the non-exudative CNV may develop more slowly [52]. This finding was also confirmed by another study, which revealed the presence of two subgroups of neovascularization, not only in BVMD but also in other retinal diseases, such as central serous chorioretinopathy and age-related macular degeneration. Authors also found that the non-exudative CNV, more stable than the exudative CNV, would seem to not require anti-VEGF injections, as they would promote atrophy progression [53].

A recent study described that CNV onset may vary based on the disease stage, ranging from nearly 30% of cases in early phases and up to almost all cases in the atrophic stage [52].

### 3.4. OCT-A in Stargardt Disease

Stargardt disease (STGD1) is one of the most frequent macular dystrophies in young adults, commonly caused by mutations in the *ABCA4* gene [54]. Its prevalence is about 1:8000–10,000 [54].

STGD1 is characterized by the loss of photoreceptors and CC, with or without the presence of yellowish lipofuscin flecks extending beyond the vascular arcades to the medium and extreme retinal periphery [55].

The clinical phenotype of STGD1 has been shown to be heterogeneous. Indeed, OCT-A was used to classify the disease phenotypes representing the disease progression, based on different choroidal patterns: pattern (1) normal choroidal thickness, few localized foveal and perifoveal yellowish–whitish flecks; pattern (2) reduced Sattler or Haller layer, numerous yellow–white fundus lesions throughout the posterior pole; pattern (3) reduced Sattler and Haller layers + extensive atrophy area; pattern (4) pattern 3 features + choroidal caverns [56].

Mastropasqua and co-authors reported, in a prospective study, the OCT-A features of 24 eyes of 12 consecutive STGD1 patients in comparison with a healthy control group [57]. A quantitative analysis was carried out, revealing a diffused vascular attenuation, especially within the foveal and parafoveal SCP and DCP, in all patients of the STGD1 group [57]. In addition, the perifoveal anastomotic arcade was interrupted in all cases to varying extents. In 15 out of 20 eyes (75%), the CC displayed the presence of well-delineated black dots, probably as an epiphenomenon of non-perfused areas [57]. The parafoveal VD of SCP was significantly lower in the STGD1 group compared to the control group (46.34 ± 4.04 vs. 52.55 ± 2.94). Foveal and parafoveal VD of the DCP were significantly lower in the STGD1 group compared to the controls (37.52 ± 9.51 vs. 29.68 ± 7.42 and 47.38 ± 4.25 vs. 59.09 ± 2.79, respectively) [57]. The same applies for foveal and parafoveal CC, both significantly lower in the STGD1 group compared to healthy eyes (54.87 ± 24.84 vs. 27.51 ± 5.37 and 60.63 ± 6.46 vs. 67.11 ± 1.40, respectively) [57].

Della Volpe et al. focused their attention on evaluating, retrospectively, the metabolic function of 107 eyes of 56 STGD1 patients, assessed with retinal oximetry, and the relation with retinal microvascular changes [58]. The authors indeed demonstrated a significant enlargement of superficial FAZ and reduced mean arterial and venular oxygen saturations in their cohort [58].

Advanced stages of STGD1 often result in macular atrophy, frequently reported as misdiagnosed in the literature [59]. An interesting study operated a comparison between the OCT-A analysis of macular atrophy in patients with atrophic STGD1 and late-stage atrophic AMD [60]. The authors reported an extensive loss of CC in the central area with persisting tissue at its margins in STGD1 patients, whereas eyes with atrophic AMD displayed an area of RPE loss with still persistent, yet rarefied CC. This finding would suggest that CC breakdown might precede outer retinal degeneration in AMD, whereas RPE and outer retinal degeneration would precede and affect CC degeneration in STGD1 [60].

### 3.5. OCT-A in Miscellaneous Diseases

#### 3.5.1. OCT-A in Gyrate Atrophy

Gyrate atrophy (GA) is an autosomal recessive chorioretinal degeneration caused by a mutation in the ornithine-δ-amino transferase (*OAT*) gene which produces a B6 enzyme that converts ornithine to glutamate [61]. GA is generally characterized by peripheral, circumferential, sharply demarcated, round patches of chorioretinal atrophy, and commonly associated with subcapsular cataract, cystoid macular edema, foveoschisis, and myopia [61]. OCT-A has been used to analyze microvascular abnormalities in patients with gyrate atrophy and cystoid macular edema. Authors reported a central dark-grey area without any evident vascular alteration attributed to a decreased signal due to the shadowing effect [62].

#### 3.5.2. OCT-A in Bietti Dystrophy

Bietti dystrophy is an autosomic recessive chorioretinal degeneration characterized by *CYP4V2* mutations, featuring yellow–white retinal and corneal crystals and progressive degeneration and atrophy of the RPE [63]. OCT-A was described as an effective tool to allow a thorough evaluation of the choroid in patients affected, as reported by Myjata et al. [63]. Indeed, authors have prospectively demonstrated CC blood flow deficit in 12 out of 13 eyes included (92%) [63]. In addition, a significant decrease in DCP and SCP in patients with Bietti disease was reported as well. [64].

#### 3.5.3. OCT-A in Leber Hereditary Optic Neuropathy

Leber hereditary optic neuropathy (LHON) is a mitochondrial inherited disorder, generally limited to the inner retina layers with characteristic loss of ganglion cells and their axons, parapapillary telangiectasia, and vascular focal tortuosity [65,66,67]. In the subacute stage of the disease, a characteristic reduction is reported in the radial peripapillary capillary density of both SCP and DCP, primarily localized in temporal sector, which corresponds to the papillomacular bundle [68,69,70]. Balducci et al., in a prospective observational study, first reported that the abovementioned microvascular changes in the temporal sector evaluated with OCT-A would be simultaneous to the GC-IPL thinning and would precede the retinal nerve fiber layer (RNFL) impairment assessed with OCT [69]. An association between the SCP and DCP vascular impairment and the RNFL reduction has also been investigated in several other published papers, which confirmed a significant association between these features, even more marked in late chronic stages [69,70,71].

#### 3.5.4. OCT-A in X-Linked Retinoschisis

X-linked juvenile retinoschisis (XLRS) is a macular degenerative disease that occurs exclusively in males and is associated with mutations in the *RS1* gene [72]. Most studies report that the schisis is mainly localized at the inner nuclear layer (INL), followed by the outer plexiform layer (OPL), the outer nuclear layer (ONL), and the ganglion cell layer (CGL) in a smaller number of cases [73]. Several studies in the literature have examined the vascular structure by the means of OCT-A, reporting a substantial enlargement and thinning of the FAZ area, telangiectasias, and vascular abnormalities at the level of both SCP and DCP, the latter of which was associated with a BCVA reduction [73,74,75,76]. Han et al. hypothesize that vascular alterations could have a primary role in the pathogenesis or may be the result of an artifact due to structural change [74].

## 4. Limitations

Imaging the retinal and choroidal layers by means of OCT-A may be challenging due to several artifacts which may confound their evaluation. Among the various source of artifacts associated with OCT-A imaging, the three that most dramatically and significantly impact the flow analysis, especially of the CC layer, include: segmentation errors, projection artifacts, and shadowing artifacts [12].

This is particularly evident in patients with IRDs due to atrophy of the outer retinal layers and RPE and to the presence of CME. The CC presents a significant segmentation challenge as it is extremely thin, and segmentation errors can cause regions of CC to be displaced outside the boundaries of the en face slab [77].

Moreover, more significant retinal vessel projection artifacts may occur in the CC in disorders with RPE disruption/atrophy.

## 5. Conclusions

OCT-A has progressively been recognized as a useful modality to evaluate retinal and choroidal blood flow in patients with IRDs. A growing body of evidence highlights its effectiveness in both diagnosis and management of these patients. Nevertheless, the role of OCT-A in the clinical management of patients with IRDs is yet to be precisely determined. Further randomized prospective studies with longer follow-ups and larger sample sizes are warranted, as they may reveal further insights into the pathogenesis and natural history of such diseases.

## 6. Methods of Literature Search

We carried out a review of literature regarding the applications of OCT-A in inherited retinal diseases using PubMed and Embase databases to November 2022 with the following terms: OCT-A in inherited retinal diseases, OCT-A in retinitis pigmentosa, OCT-A in choroideremia, OCT-A in Best disease, OCT-A in Stargardt disease, OCT-A in gyrate atrophy, OCT-A in Bietti Dystrophy, OCT-A in Leber Hereditary Optic Neuropathy, OCT-A in X-linked Retinoschisis, and combination of these. All relevant publications written in English were sourced, including prospective and retrospective clinical studies, and laboratory experimental studies. We included case reports only if they contributed new and relevant information about applications of OCT-A in inherited retinal diseases.

## Figures and Tables

**Table 1:** Optical coherence tomography angiography features in patients with retinitis pigmentosa.

Authors	Study	F-UP	N. Eyes	SCP VD	DCP VD	CC VD	CH VD	ONH/RPL VD	FAZ Area
Alnawaiseh [15]	P	NA	20	Reduced	Reduced	Reduced	/	Reduced	Increased
Arrigo [16]	P	12 MO	68	Reduced	Reduced	/	/	/	/
Atas [17]	R	NA	26	Reduced	Reduced	/	/	/	/
Attaallah [18]	P	3 MO	24	Reduced	Reduced	Reduced	/	/	Increased
Deutsch [19]	R	24 MO	29	Reduced	Reduced	Reduced	Reduced	/	Reduced
Giansanti [20]	R	13 MO	52	Reduced	Reduced	Reduced	Reduced	/	Reduced
Hagag [21]	P	NA	44	Reduced	Reduced	/	/	/	/
Jauregui [22]	R	15 MO	28	Reduced	Reduced	/	/	/	Increased
Koyanagi [23]	R	24 MO	73	Both Reduced	Both Reduced	/	/	/	Increased
Liu [24]	R	36 MO	53	/	/	Reduced	Reduced	/	/
Mastropasqua [25]	P	6 MO	20	Both Reduced	Both Reduced	Both Reduced	/	/	/
Miyata [26]	P	2 MO	43	/	/	Reduced	/	/	/
Nakajima [27]	R	NA	38	/	Reduced	/	/	Reduced	/
Nassisi [28]	R	9 MO	28	Reduced	Reduced	Reduced	/	/	/
Parodi [29]	R	8 MO	32	Reduced	Reduced	/	/	/	Increased
Shen [30]	P	10 MO	34	Reduced	Reduced	/	/	/	/
Sugahara [31]	R	NA	68	Reduced	Reduced	/	/	/	/
Takagi [32]	R	6 MO	50	Reduced	Reduced	/	/	/	Reduced
Toto [33]	R	NA	28	Reduced	Reduced	Reduced	/	/	/
Wang [34]	P	NA	40	Both Reduced	Both Reduced	Both Reduced			Increased

CC: choriocapillaris; CVI: choroidal vascularity index; CH: choroid; DCP: deep capillary plexus; F-UP: follow-up; FAZ: foveal avascular zone; MO: months; NA: not applicable; N.: number of; ONH: optic nerve head; P: prospective; R: retrospective; RPL: radial peripapillary layer; SCP: superficial capillary plexus; VD: vessel density. Results were significant for *p* < 0.05.

## Data Availability

Data sharing is not applicable to this article as no datasets were generated or analyzed in the current article.
